# The Differential Impact of Lockdown Measures Upon Migrant and Female Psychiatric Patients – A Cross-Sectional Survey in a Psychiatric Hospital in Berlin, Germany

**DOI:** 10.3389/fpsyt.2021.642784

**Published:** 2021-05-28

**Authors:** James K. Moran, Joachim Bretz, Johanna Winkler, Stefan Gutwinski, Eva J. Brandl, Meryam Schouler-Ocak

**Affiliations:** Department of Psychiatry and Psychotherapy, St. Hedwig Hospital, Charité-Universitätsmedizin Berlin, Berlin, Germany

**Keywords:** COVID-19, lockdown measures, migrant psychiatric patient, cultural psychiatry, survey

## Abstract

The COVID-19 pandemic could have major effects on already vulnerable individuals with psychiatric disorders. It is important to assess how different patient groups respond to stress related to the pandemic, and what additional factors influence it, including family-related stress, migration background, and sex. We conducted a survey in a sample of 294 psychiatric patients in a large outpatient clinic in Berlin, measuring level of distress in relation to COVID-19 lockdown as well as family-related distress. We also measured potential influencing factors such as media consumption and medical support. In the migration background group, we found that women had more lockdown related psychological distress than men. This was not apparent in those patients with a German background. We found that females were more strongly affected by family-related distress, particularly those with a migration background. People with PTSD were most strongly affected by family-related distress, whereas people with psychotic disorders and addiction reported the least distress. There were no effects of media consumption. There were no differences in ability to abide by the lockdown related restrictions across diagnoses. Our results support earlier findings on differential vulnerability of diagnostic groups to these stressors. Thus, clinicians can optimize treatment by taking family-related stressors into account particularly for females and people with a migrant background.

## Introduction

The COVID-19 crisis has necessitated radical society-wide interventions to limit the spread of the SARS-CoV-2 virus. Some of these are comparatively minor – enforcing stricter hygiene and masks, others required major readjustments to everyday life. In particular periods of “lockdown” that first took place for the first time in many countries between March and June 2020. In Germany at this time, this involved a series of escalating restrictions to everyday life, including limitations on gatherings (meeting with maximum 1 person other than people you live with, in open air, legal penalty for large gatherings such as parties), travel restrictions (only essential travel overseas or within Germany permitted), minimization of movement outside of the house (limited essential things such as shopping, doctor appointments), restaurants, bars, retailers were closed (1). The isolation and stress appear to have had a psychological affected the general population, with higher depression, anxiety, and stress related disorders (2).

To date, studies on the responses of psychiatric patients to the pandemic conditions suggest that psychiatric patients are more strongly affected by strict lockdown measures in comparison to healthy controls, including insomnia, PTSD symptoms, and suicide ideation (3). Another study found a generally low level of knowledge of COVID-19, lower compliance with health regulations and a lower level of anxiety regarding COVID-19 in comparison to the general population (4). Attempts to test the differential impact of the pandemic suggest that people with affective disorders are far more affected by the restrictions imposed by COVID-19, whereas people with psychotic disorders are less affected (5, 6) Specific population groups may be especially vulnerable to stress resulting from the COVID-19 crisis. Psychiatric patients have more precarious employment (7); smaller social networks (8); lower health literacy (9); greater physical vulnerability via higher rates of obesity (10), and smoking (11, 12). Moreover, lockdown and quarantine could exacerbate symptoms via loneliness, isolation and double stigma (8). Studies further suggest that certain groups of psychiatric patients may have greater difficulty adhering to the various restrictions such as social distancing, and stricter hygiene, via reduced executive function (12), maladaptive denial of illness (13), psychotic interpretation of the illness (14).

The potential vulnerability of psychiatric patients to the burden of lockdown measures is compounded by other demographic factors, in particular migration background. People with a migration background show generally worse health outcomes (15), this applies more specifically to anxiety disorders, including PTSD (16), depression (17), psychosis (18, 19) and suicide mortality (20, 21). Effects of psychiatric problems can be intergenerational, for example, traumatized survivors of war can have a harsh punitive parenting style, leading to greater aggression, parental depression and anxiety affects early attachment (22). Subsequent generations of migrants are thus correspondingly at higher risk of poor health outcomes (23, 24). There is evidence to suggest that ethnic minorities are disproportionately affected by the current pandemic (25, 26), partly as worse overall health outcomes in certain populations (e.g., African Americans with greater hypertension, and diabetes), and likelihood of living in high-density housing. The greater risk of the getting the virus could increase anxiety in the lockdown situation.

There are also differences in health outcomes for females and males. In the context of COVID-19, it appears that males are more strongly affected by contraction of the virus itself (27), which may lead to a higher level of anxiety during lockdown. In contrast, the indirect consequences could potentially affect females more due to their greater socioeconomic vulnerability (28). In particular, there are indications of increased domestic violence (29, 30).

Certain other factors also potentially influence the impact of COVID-19 on psychiatric patients. For example, increased media exposure relating to COVID-19 predicts greater distress in psychiatric patients (31, 32). In contrast, adequate support can have a protective effect (33, 34).

The present study examines the experience of the pandemic for a cohort of outpatients at a large psychiatric outpatient clinic in Berlin, Germany, with a high percentage of patients with a migration background. This diverse sample provides an opportunity to examine the effect of the pandemic on different diagnostic groups, as well as across migrant background and gender. Furthermore, the effects of the pandemic on family life and the influence of the media are also measured. We also test whether there were differences in the ability to comply with hygiene and other lockdown measures across these different groups.

## Methods

### Participants

From April 1st 2020 to June 30th 2020, we invited patients at the outpatient psychiatric clinic at the Psychiatric University Hospital of the Charité at St Hedwig hospital, Berlin, Germany, who did not fulfill exclusion criteria to participate in the study (acute suicidality, severe cognitive impairment). The clinic treats around 1,500 patient per quarter at its branches. Of these, we recruited a sample of *N* = 294 patients. The survey was administered in the context of an ordinary consultation, where clinicians' sometimes work under time pressure and the patients' own priorities are often focused on their own acute needs in the moment. This meant that not all of the potential pool of patients could be offered the survey, accounting in part for the relatively small proportion of respondents. The survey was conceived to be conducted in interview form by all staff of the clinic after a training session (nurses, psychologists, physicians, social workers). However, the survey could also be taken home to be filled in by the patient and brought back later in the same quarter. Sometimes the first few questions were administered in interview form, and the patient was asked to complete the questionnaire after the consultation. Telephone consultations accounted for 47% of the sample. If needed, the interviews were done in the native language of migrant patients via interpreters. The study was approved by the Ethics Committee of the Charité, Universitätsmedizin Berlin (EA4/251/19).

The statistical analyses were carried out with R 3.6.3. For ANOVA, Kruskall-Wallace tests with follow up Dunn's tests for contrasts were used where assumptions were not fulfilled [homogeneity of variance (Levene's test), normality (Shapiro-Wilk), extreme outliers (>|4| SD)]. *Post-hoc* contrasts were corrected for multiple comparisons using Bonferroni or Benjamini-Hochberg corrections. For crosstabulation statistics, we used either χ^2^-square statistics or Fischer exact tests, according to the numbers in the cell sizes. Where the null hypothesis of independence was not supported, we examined the deviation of cell numbers from standardized residuals, adjusting the threshold of significance against the number of comparisons with Bonferroni contrasts. Where the interpretation of a null finding was relevant, we computed we computed Bayes Factors [BF10, (35)] as an indicator of the relative evidence for the H0 and H1. BFs between 1 and 3 indicate anecdotal support for the alternative hypothesis (H1) while BF between 3 and 10 and above 10 indicate respectively moderate and strong support for H1. BF = 1 indicates equal support for H1 and null hypothesis (H0) while BF between 1/3–1, 1/10–1/3, and below 1/10, provide respectively anecdotal, moderate, and strong support for H0 (36).

Because we were interested in the way that people with a migration background were possibly affected by the COVID-19 crisis, we distinguished between people with a German background, and those with a migrant background. The latter is defined as people who were either born outside of Germany, or who are born from parents who migrated to Germany.

### Survey Development

Potentially relevant demographic variables were collected, including age, sex, ICD-10 diagnosis, daily hours of media consumed, and details of migration background where applicable, including number of years in Germany. We generated a questionnaire on the basis of our experiences with patients during COVID-19. We asked about the different potential ways in which COVID-19 could affect individuals (fear for loved ones, sleep, physical complaints, anxiety, worsening symptoms, and drug consumption, fear of contagion). These were classified in a binary manner (yes/no), and then summed to make the variable “Lockdown related distress,” with a potential score range of 0–7). We also measured family-related distress in the same way (more household tension, more work, overwhelmed, more arguments, more domestic violence), to create the outcome variable “Family-related distress,” with a potential score range of 0–5. Additionally, we asked whether other factors such as news media consumption and the feeling that they have a doctor that understands their problems and supports them, affected the state of patients.

## Results

Demographic variables, including age and sex, years spent in Germany, diagnosis, migration background, country of origin, daily hours of media consumption, years in Germany, current family living arrangements, contact type, and reason for contact, are shown in Table 1. For the subsequent analyses of the specific diagnostic groups, we grouped disorders with small numbers of affected patients. Because of a large number of missing responses, the number participants for the different analyses ranged from 221 to 294. Because the questionnaire is not based on a standardized questionnaire with e.g., an established factor structure, we did not use a data imputation strategy.

**Table 1:** Demographic information for survey participants missing data for each group are included.

**Variable (*N*, missing)**		
Age (280,14)	mean	
M *(SD)* [Range]	44.61 *(12.85)* [18–78]	
Sex (279, 15)	female	male
*N (%)*	153 *(54.8)*	126 *(45.2)*
Migration background (280,14)	German background	Migration background
*N (%)*	135 *(48.2)*	145 *(51.8)*
Country *N (%)*		Turkey 55 (*19.6*) Afghanistan 17 (*6.1*)
		Syria 7 (*2.5*)
		Lebanon 5 (*1.8*)
		Iraq 4 (*1.4*)
		Iran 4 (*1.4*)
		Other 53 (*18.9*)
Migrant years in Germany (125, 20)M *(SD)* [Range]	20 *(16.86)* [1–75]	
Hours of media consumed per day (263, 31)M *(SD)* [Range]	1.37 *(1.78)* [0–12]	
living alone (282,12)	with others	alone
*N (%)*	148 *(52.5)*	134 *(47.5)*
living with family (267,27)	with family	other
*N (%)*	126 *(47.2)*	141 *(52.8)*
Diagnosis (248, 46) *N (%)*	F1 Addiction	8 *(3.2)*
	F2 Psychotic disorder	45 *(18.1)*
	F3 Mood disorder	101 *(40.7)*
	F43 trauma related disorder	18 *(7.3)*
	F4 40 41 anxiety disorder	37 *(14.9)*
	F4 44, 45 other neurotic disorder	12 *(4.8)*
	F6 personality disorder	10 *(4)*
	F0, F42, F5, F7, F8, F9 other	17 *(6.8)*
Contact	Regular appointment 148 spontaneous 137	
	Telephone 135 Face to face 150 Interpreter 11	
Reason	Prescription 56 Need to talk 145 Crisis 1 Other 54	

Family-related distress stemming from COVID-19 restrictions are outlined in Table 2A. A greater general level of tension in the household was reported by the majority of patients (56.49%), with similarly high amounts of house work (54.85%). A small minority of people reported greater household violence (4.42%) (a follow up analysis of the last including only those people living with others (*N* = 148) showed a similar rate 4.41%). Table 2B shows overall outcomes for lockdown related distress, this encompasses psychological responses to COVID-19. A majority of patients reported worry about relatives (54.22%) and anxiety (52.10%), worse sleep (44.04%), more physical symptoms (35.99%) and a small minority reported increased drug consumption (13.15%). Table 2C shows responses to restrictions, suggesting close to ceiling level effects in regard to the ability to maintain hygiene, social-distancing, and stay informed. Patients were also invited to provide qualitative responses to the questions, some illustrative examples are provided.

**Table 2:** Responses to the survey questions grouped into three themes from left to right: family related stress, lockdown related stress, and ability to cope with restrictions.

**(A) Family-related distress:**		
How do you cope in the family with the restrictions (children at home, school and kindergarten, partner at home)?	Example of qualitative responses	
%		
more household tension	56.49	“my mother is fearful”
work problems	54.85	“my husband lost his job, that worries me”
overwhelmed	35.78	“worried that the kids would catch the virus”
more arguments	23.18	“everyday household stuff, like cleanliness”
more domestic violence	4.42	“no physical aggression, but more verbal aggression”
**(B) Lockdown-related stress:**		
How are you coping with the Corona Crisis?		
	%	
worry about relatives overseas	54.22	“my mother, she has a condition that makes her vulnerable”
higher anxiety	52.10	“fear that all groceries will be sold out”
more psychological problems	48.10	“fear – I don't go out at all anymore”
worse sleep	44.04	“not enough exercise, disturbed day/night rhythm”
fear of catching the virus	39.07	“frightened of not getting a spot in hospital”
physical complaints	35.99	“exhaustion from brooding on the situation”
more drugs (alcohol, sedatives, cocaine, LSD, etc.)	13.15	“I smoke more at the moment”
**(C) Coping with restrictions**		
	%	
do you feel well-informed?	86.22	“yeah, too much, I avoid the news and social media”
adhere to hygiene recommendations?	96.19	“I wash my hands but forget the mask and have to buy them when I'm out and about”
can you maintain social distancing?	89.82	“yes, but it's difficult on public transport”
Understanding doctor	67.40	“yes but the practice is large, with many different doctors”
more media consumption?	58.25	“at the beginning yes, now I avoid it”
more news?	56.21	“more TV, social media, otherwise talk with friends”

*Percentages represent the percentage of affirmative responses across the whole sample*.

These two sets of questions were combined into two numeric variables, family-related stress, and lockdown-related distress. Firstly, there was no association between the overall amount of family-related distress resulting from lockdown measures and the amount of media consumed [*F*_(1, 261)_ = 0.027 *p* = 0.870]. Similarly, there was no relation between the amount of individual stress and amount of media consumed [*F*_(1, 261)_ = 1.19 *p* = 0.276], or the presence of an understanding doctor [*F*_(1, 271)_ = 0.07, *p* = 0.787]. To test for response bias, we analyzed whether there were differences in responses to the two outcome variables (family-related stress, and COVID distress) according to contact type (face-to-face, telephone, interpreter, regular appointment, spontaneous) as well as reason for appointment (prescription, need to talk, crisis, other). There were no differences across these response bias variables (all *p* < 0.05). Subsequent BF were all < 0.333, indicating strong support for the null hypothesis. One exception was the relation between reason for consultation and family-related distress, which at 1.04, indicated no clear distinction between H0 and H1.

**Figure 1:**
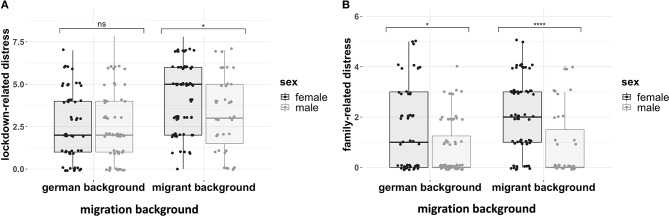
**(A)** Lockdown-related distress across sex and migration background. **(B)** Family related distress across sex and migration background. Both boxplots show interquartile range, with the middle bar being the median value. *N* = 221. Significance bars refer to follow-up Bonferroni-adjusted contrasts (****p < 0.00001, *p < 0.05, ns = non-significant).

To test whether lockdown-related stress varied across sex, and migration background a factorial ANOVA was run. There were main effects of migration background (see Table 3A and Figure 1A), suggesting that ~9.8% of variance in lockdown-related stress was related to migration background. Follow-up Bonferroni adjusted *post-hoc* contrasts showed that females with a migrant background had much higher levels of family pressure than males (Adj-mean difference = 1.00, *p*_bonf_ = 0.037). Amongst the people with a German background, there was no difference between males and females (Adj-Mean difference = −0.094, *p*_bonf_ = 1.00), see Table 3A.

**Table 3:** ANOVA table for (A) Lockdown related stress against migration background and sex. (B) Family related pressure against migration background and sex.

**(A) Lockdown related distress**	***F*_**(1, 217)**_**	**η^**2**^**	**(B) Family related distress**	***F*_**(1, 217)**_**	**η^**2**^**
Migration background	23.56^****^	0.098	Migration background	8.16^**^	0.036
Sex	2.06	0.009	Sex	23.21^****^	0.097
Sex*migration background	3.62	0.016	Sex*migration background	1.64	0.007

*Effect size = partial η^2^*.

** *p < 0.01*.

**** *p < 0.0001*.

To test whether family-related distress varied across sex, and migration background a factorial ANOVA was run. There were main effects of both migration background and sex (see Table 3B and Figure 1B). suggesting that ~9.7% of variance in lockdown-related stress was related to sex, and 3.6% was attributable to migration background. Follow-up Bonferroni adjusted *post-hoc* contrasts showed that females from a migrant background had much higher levels of family related stress than males (Adj-mean difference = 1.2, *p*_bonf_ = 0.0001). Amongst the people with a German background, the difference was also apparent but less pronounced (Adj-Mean difference = 0.69, p_bonf_ = 0.020), see Table 3B.

### Lockdown Related Distress by Diagnosis

A non-parametric Kruskal-Wallis test showed that there was variation across different diagnostic categories for lockdown-related distress [χ^2^ (7) = 16.82, *p* = 0.019]. Follow-up pairwise contrasts, corrected with Benjamini-Hochberg corrections, could not more precisely specify the difference between the diagnostic groups. Since there could be systematic differences across gender and migration background, we repeated the analysis separating them into groups. None of the analyses were significant.

### Family-Related Distress by Diagnosis

**Figure 2:**
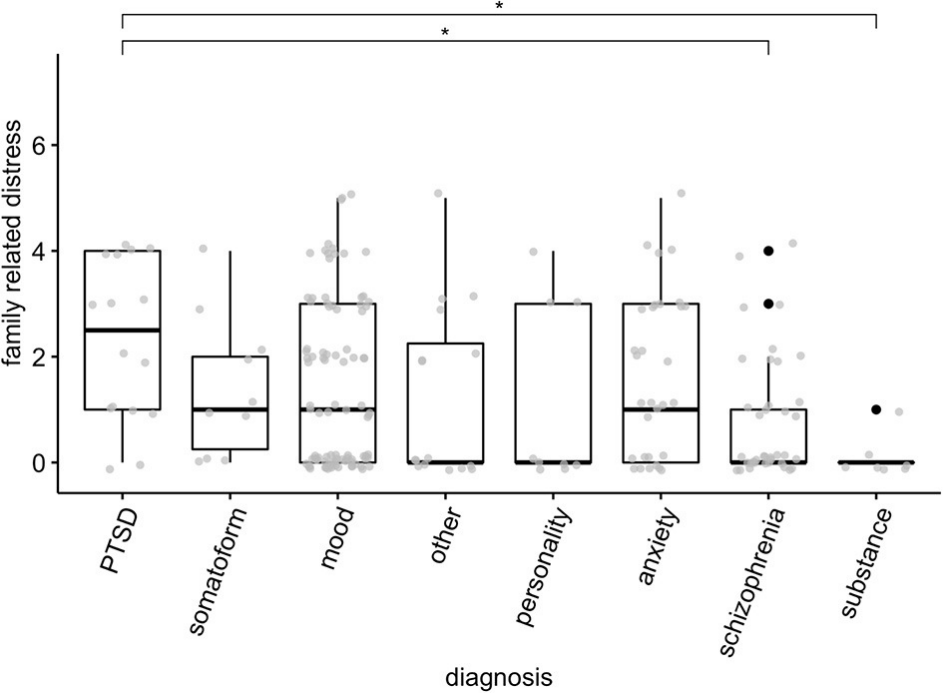
Family related distress across different diagnoses. Boxes represent quartiles, black dots represent outliers. Gray points represent scores of individual patients. *p < 0.05.

A non-parametric Kruskal-Wallis test showed that there was variation across different diagnostic categories for family-related distress [χ^2^ (7) = 19.32, *p* = 0.007] (Figure 2). Follow-up pairwise contrasts, corrected with Benjamini-Hochberg corrections, suggested that group with the highest level of family related stress – PTSD – differed significantly from those groups with the lowest family-related distress, namely schizophrenia (*Z* = 3.40, *p*_bh_ = 0.019), and substance-use disorders (*Z* = 3.35, *p*_bh_ = 0.011). Since there could be systematic differences across gender and migration background, we repeated the analysis separating them into groups. None of these analyses showed significant differences.

### Migrant Responses to COVID-19 Measures

We asked patients how they were coping with the various restrictions relating to COVID-19 and whether this varied across gender or migration background. The overall responses for all patients are set out in Table 2C. These factors were individually examined for potential differences between gender and migration background using separate logistic regressions: how well-informed participants felt regarding COVID-19, whether they felt able to take precautionary measures (e.g., hand-washing, mask wearing), and how well they were able to maintain social-distance. A male with a German background was less than half as likely to report being able to maintain social distance compared to a male with a migration background (OR = 0.44, *z* = 1.05, *p* = 0.028). Otherwise, there were no differences across gender or migration background regarding how well-informed they felt or their capacity to take precautionary measures, or whether they felt understood by their doctors (all *p*-values > 0.05).

## Discussion

We found differences in response to family-related stress as well as lockdown-related stress according to sex, migration background and diagnostic group in psychiatric outpatients. We could not find evidence of difficulty holding to the new guidelines among our patients or any relation to hours media consumption.

Our sample reported higher levels of tension and stress in the household, only a small number of patients reported greater levels of domestic violence. Follow-up analysis showed that females reported greatest levels of family-related stress. This was valid across both participants with and without a migration background, although the effect on those with a migration background was stronger. This is perhaps because they more often have children than female outpatients without a migrant background (37). This supports hypotheses that females were more affected by the socioeconomic aspects of the pandemic (28). The in 4.42% reported cases of increased domestic violence is on the face of it, different to those reporting far higher prevalence (29, 30). It should be emphasized that this represents a reported relative increase, rather than an absolute amount of domestic violence. Results did not differ when excluding those who lived alone (4.41%). In the case of sensitive questions such as this, the influence of social desirability or shame in reporting domestic violence cannot be ruled out (38).

It appears that females with a migrant background showed greater individual negative effects in regard to Lockdown related stress. Thus, the higher risk of bad outcomes for men from the SARS-CoV-2 virus itself (28), does not translate into higher anxiety or stress levels. More research is needed to examine why women with a migrant background are particularly affected.

Earlier studies suggested different responses to external stressors in patients with different psychiatric disorders. For example, people with anxiety or depression were reported to respond with greater levels of decompensation to external stress, whereas those with psychosis were less affected by external stress (5, 31). Our results partly bear this out, with people with PTSD showing the highest level of lockdown related distress, and those with psychotic disorders showing the lowest levels of lockdown related distress. Patients with mood disorders trended in the same direction of greater distress as well but the difference was not significant. It is possible that patients with psychotic disorders, particularly schizophrenia, are driven more by endogenous factors with less influence from the outside (5). However, there are complex relations between social factors, and loneliness in serious mental illness (SMI) (39). For example, although endogenous symptoms appear to be the strongest determinants of quality of life in people with serious mental illness (SMI), (40), this is mediated by support from family and friends (40, 41). It is possible that their illness already exerts a severe baseline restriction on their quality of life, and therefore the effects of the pandemic are judged less severe in this context. Reports of our patients with psychotic disorder point in this direction, because they sometimes reported in the last months that they are experts in isolation, because they are always isolated, in contrast to the whole population for whom such restrictions are more novel. There was no interaction of gender and diagnosis or migration and diagnosis on either lockdown related distress or family related problems.

An earlier study found lower compliance with health regulations in psychiatric patients in comparison with healthy controls (4). In our study, the majority of patients reported feeling well-informed, and being able to comply with lockdown, social-distancing, and hygiene measures. It is possible that the patients refusing these regulations were also more likely to refuse participation in this survey. We have no healthy control comparison, so we cannot draw a general conclusion, however our results did tend toward ceiling effects (feeling well-informed, 86%; adhering to hygiene measures, 96%), thus making a major difference between psychiatric patients and healthy controls less probable.

We found no influence of media consumption upon subjectively experienced lockdown distress or family-related distress. This contrasts with other findings, showing that increased media exposure relating to COVID-19 predicts greater distress in psychiatric patients (31, 32). There could be several reasons for our null results in comparison to those of other researchers, e.g., differences between outpatient vs. inpatients, our clinic's focus of culturally sensitive psychiatric treatment, or differences between countries and cultures and relative impact of COVID-19. Clarifying this requires more focused research on this issue comparing potential factors across cultures.

Our study had several limitations. Given the novelty of the pandemic situation, we opted to tailor our survey questions directly to the situation, rather than adapt pre-existing questionnaires, which however has a cost in terms of reliably comparability to other findings. Our response rate was comparatively low, possibly introducing a selection bias into our data, e.g., perhaps the more conscientious patients or less crisis-stricken patients were more likely to take part. The decision to allow the patient to complete the survey at home was necessitated by time constraints in the outpatient clinic. We cannot exclude the possibility of response biases arising from this, although we found no evidence for differences in survey administration in our outcome variables. A mixed model would have been ideal means of considering the complex interactions of the different factors, however, the data did not fulfill necessary assumptions. We therefore employed simpler robust statistics with appropriate correction for multiple comparisons. Our null results, which stand in contrast to some other results need to be interpreted with the caution that an “absence of evidence is not evidence of absence.”

Overall, our results are in agreement with other studies showing a greater impact of lockdown related stress on people with PTSD, and less effects on people with schizophrenia. Females show greater lockdown related distress as well as family-related distress. The latter is further exacerbated in those patients with a migration background. In which case, it would suggest the need for clinicians and clinics to take more consideration of the family situation in psychiatric outpatients during the pandemic, with a particular focus on people with a migrant background.

## Data Availability Statement

The datasets are available in a de-identified form from the corresponding author upon reasonable request.

## Ethics Statement

The studies involving human participants were reviewed and approved by Ethics Committee of the Charité, Universitätsmedizin Berlin (EA4/251/19). The patients/participants provided their written informed consent to participate in this study.

## Author Contributions

JM, JB, and EB: analysis. All authors study design, implementation, and manuscript preparation.

## Conflict of Interest

The authors declare that the research was conducted in the absence of any commercial or financial relationships that could be construed as a potential conflict of interest. The reviewer SW declared a shared affiliation, though no other collaboration, with the authors JM, JB, JW, SG, EB, MS-O, to the handling editor.
