# Supplementary material for: The Differential Impact of Lockdown Measures Upon Migrant and Female Psychiatric Patients – A Cross-Sectional Survey in a Psychiatric Hospital in Berlin, Germany
Source: Front Psychiatry. 2021 May 28;12:642784. doi: 10.3389/fpsyt.2021.642784 (PMC8192805; doi:10.3389/fpsyt.2021.642784)
Supplement: Supplementary file 1 [file Data_Sheet_1.DOCX]

**Effects of social distancing and lockdown restrictions during the Coronavirus Pandemic on social cohesion within families.**

**Code: ________ (Surname first letter + Date of birth e.g. K02031994)**

**Questions:**

1. **Contact (Please check, if apply)**

- Regular agreed appointment
- By phone
- Face-to-Face
- with Interpreter ________________________

1. **Reason for contact: (please check, if applicable)**

- Need a prescription
- Need to talk
- Decompensation/crisis

What specifically? (Please describe in brief):

____________________________________________________________________________________________________________________________________________________________________________________________________________

o Other Reasons (Please describe in brief):

____________________________________________________________________________________________________________________________________________________________________________________________________________

1. **Socio-demographic characteristics:**

- Age ____
- Sex
  - male
  - female
  - diverse
- living alone
  - yes
  - no
- with family
  - yes
  - no
- How many people are living in your household? ____
- In your own apartment?
  - yes
  - no
- An home?
  - yes
  - no
- Anything else:

____________________________________________________________________________________________________________________________________________________________________________________________________________

- Country of origin ________________________________
- Duration of stay in Germany_____________
- Language skills _____________________________
- Diagnosis

____________________________________________________________________________________________________________________________________________________________________________________________________________

1. **Do you have other contacts in the time of Corona? To**

- Acquaintances
  - yes
  - no
- Neighbours
  - yes
  - no
- Friends
  - yes
  - no
- Another person
  - yes
  - no

1. **Do you feel well-informed about the corona restrictions?**

- yes
- no
- details

__________________________________________________________________________________________________________________________________________________________________________________________

1. **Can you put the recommended measures into practice, such as washing and disinfecting your hands regularly?**

- yes
- no
- details

__________________________________________________________________________________________________________________________________________________________________________________________

1. **Is it possible for you to keep the recommended distance?**
   - - yes
     - no
     - details

__________________________________________________________________________________________________________________________________________________________________________________________

1. **How do you cope with the lockdown restrictions?**

- More media consumption
- yes
- no
- details

__________________________________________________________________________________________________________________________________________________________________________________________

- More news than usual
  - - yes
    - no
    - details

__________________________________________________________________________________________________________________________________________________________________________________________

- What sources do you use to get your news?

____________________________________________________________________________________________________________________________________________________________________________________________________________

- How much time do you spend with news every day?

________________________________________________________________________________________________________________________________________

____________________________________________________________________

- Do you use more media from your country of origin or in your native language?
  - - yes
    - no
    - details

__________________________________________________________________________________________________________________________________________________________________________________________

1. **In your family, how do you cope with the lockdown restrictions (Children at home, School and Kindergarten, Partner at Home)?**

- Feeling overwhelmed
  - - yes
    - no
    - details

__________________________________________________________________________________________________________________________________________________________________________________________

- Feeling more burdened than usual
  - - yes
    - no
    - details

__________________________________________________________________________________________________________________________________________________________________________________________

- Feeling more stressed than usual
  - - yes
    - no
    - details

__________________________________________________________________________________________________________________________________________________________________________________________

- More conflicts in my family than usual
- yes
- no
- details

__________________________________________________________________________________________________________________________________________________________________________________________

- More violence
  - - yes
    - no
    - details

__________________________________________________________________________________________________________________________________________________________________________________________

- - - Anything else

__________________________________________________________________________________________________________________________________________________________________________________________

1. **How are you feeling about Corona crisis?**

- More worries than usual:
- yes
- no
- details

__________________________________________________________________________________________________________________________________________________________________________________________

- Worrying more about relatives in my country of origin?
- yes
- no
- details

__________________________________________________________________________________________________________________________________________________________________________________________

- Fear:
- yes
- no
- details

__________________________________________________________________________________________________________________________________________________________________________________________

- Sleep disorders:
- yes
- no
- details

__________________________________________________________________________________________________________________________________________________________________________________________

- physical complaints:
- yes
  - if yes, which ones:

_____________________________________________________________________________________________________________________________________________________________________

- no
- more psychological complaints
- yes
  - if yes, which ones:

_____________________________________________________________________________________________________________________________________________________________________

- no
- more drugs (Alcohol, tranquilizers, cocaine, LSD…)
- yes
  - if yes, which ones:

_____________________________________________________________________________________________________________________________________________________________________

- no

1. **What helps you to better endure this whole situation?**

________________________________________________________________________________________________________________________________________________________________________

1. **Are you very afraid of getting sick with the Corona virus infection?**
   - - - yes
       - no
       - details

__________________________________________________________________________________________________________________________________________________________________________________________

1. **Do you have a primary care doctor (who you feel understands you)?**

- yes
- no
- details

__________________________________________________________________________________________________________________________________________________________________________________________

1. **Who can you turn to with questions about Corona?**

_______________________________________________________________________________________________________________________________________________________________________________________________________________________________________

1. **Is there anything else you would like to say?**

__________________________________________________________________________________________________________________________________________________________________________________________________________________________________________

Thank you very much for your trust!
